# *Cayratia japonica* Prevents Ulcerative Colitis by Promoting M2 Macrophage Polarization through Blocking the TLR4/MAPK/NF-*κ*B Pathway

**DOI:** 10.1155/2022/1108569

**Published:** 2022-12-30

**Authors:** Jun Sun, Ping Zhao, Xufeng Ding, Fang Li, Jie Jiang, Hua Huang, Lijiang Ji

**Affiliations:** ^1^Department of General Surgery, Jingjiang People's Hospital, Jingjiang 214500, China; ^2^Department of Anorectal, Changshu Hospital Affiliated to Nanjing University of Chinese Medicine, Changshu 215500, China; ^3^Department of Gastroenterology, Changshu Hospital Affiliated to Nanjing University of Chinese Medicine, Changshu 215500, China

## Abstract

**Background and Aims:**

Several components of *Cayratia japonica* (CJ) such as rutin and quercetin have shown anti-inflammatory effect, yet its function in ulcerative colitis (UC) remains to be clarified. This study focuses on the modulatory effect of CJ on UC as well as molecular mechanism by which CJ regulates macrophage polarization in UC.

**Methods:**

The targets related to CJ components and UC were, respectively, obtained through *in silico* analysis, and their intersection targets were selected for pathway enrichment analysis. RAW264.7 cells were stimulated with lipopolysaccharide (LPS) to induce M1 macrophages. Expression of the macrophage polarization M1 marker CD11b and M2 marker CD206 was measured to determine the phenotype of macrophages. The mouse model was treated with dextran sodium sulfate (DSS) to induce UC to observe the effects of CJ on UC *in vivo*.

**Results:**

The *in silico* analysis suggested the crucial significance of TLR4 and its downstream MAPK/NF-*κ*B pathways in the modulatory effect of CJ on UC. Furthermore, experimental data revealed that CJ could promote M2 macrophage polarization but alleviate immune inflammation and reduce colon damage in DSS-evoked UC model. Additionally, CJ can inhibit the expression of TLR4/MAPK/NF-*κ*B signaling pathway to enhance the M2-like polarization.

**Conclusion:**

Hence, CJ may exert anti-inflammatory effects and an inhibitory role in UC by inhibiting the TLR4/MAPK/NF-*κ*B pathway and subsequent M1-like macrophage polarization.

## 1. Background

Ulcerative colitis (UC) affects millions of people worldwide, which is featured by extensive colonic injury including mucosal and submucosal layers of the colon [1, 2]. It is mainly clinically characterized by diarrhea, abdominal pain, bloody purulent stool, and tenesmus, which truly affects the quality of life for patients [3]. Genetic and environmental factors are reported to affect the initiation of UC [4]. Most UC cases receive pharmacological therapy to first induce remission and later to maintain a corticosteroid-free remission, which requires different kinds of drugs used, such as the oral or rectal administration of 5-aminosalycilates (5-ASA) [5]. However, given the increasing incidence of UC, identification of novel therapeutic targets is ongoing for UC treatment [6].

Traditional Chinese medicine (TCM) bears great responsibility for UC treatment [7, 8]; for instance, Guchang Zhixie Wan is a commonly used TCM for the treatment of UC [9]. *Cayratia japonica* (CJ) is a perennial vine belonging to *Vitaceae* and has been wildly applied as a folk medicine in China to treat jaundice, diarrhea, edema, rheumatalgia, erysipelas, and hematuria [10]. However, the role of CJ in UC is rarely reported in the existing literature, which warrants further exploration. Two major components of CJ, rutin and quercetin, can be used in the treatment of inflammatory bowel diseases [11]. Rutin can also restrain lipopolysaccharide- (LPS-) induced macrophage inflammation via the inhibition of the Toll-like receptor 4- (TLR4-) NF-*κ*B signaling [12]. Furthermore, a study has unveiled that TLR4 alone shares an association with UC [13]. Another study has revealed rutin and quercetin to be the components of *Zanthoxylum bungeanum* pericarp extract, which ameliorates experimental colitis through modulating the TLR4 and TLR4-associated pathway [14]. Moreover, overexpression of TLR4 may induce the formation of cellular microenvironment supporting tumor growth and accelerate colitis-associated tumorigenesis [15]. The TLR4/NF-*κ*B signaling pathway can induce macrophage activation and acts importantly in the acute inflammation of LPS induced by macrophages [16, 17]. Collectively, our study conducted network pharmacology and cell experimentations to analyze the pharmacological mechanism of CJ for UC treatment.

## 2. Materials and Methods

### 2.1. Drug Target Acquisition

Through the PubChem database (https://pubchem.ncbi.nlm.nih.gov/), the interactive chemical structure model of rutin, chlorogenic acid, and quercetin was retrieved separately, and 3D conformer was downloaded as the pharmacophore, which was then uploaded to the PharmMapper database (http://www.lilab-ecust.cn/pharmmapper/) to predict the targets. Through UniProtKB database (https://www.uniprot.org/), the official gene symbol corresponding to the target (limited to “*Homo sapiens*”) was retrieved. From the CTD database (http://ctdbase.org/), the corresponding targets of rutin, chlorogenic acid, and quercetin were obtained.

### 2.2. Dataset Analysis for Disease Target Identification

We screened 2 UC-related microarray datasets (GSE48958 and GSE65114) from the GEO database. The GSE48958 dataset contained colon mucosa samples from 8 normal people and 7 active UC patients, which was equipped with platform annotation file GPL6244. The GSE65114 dataset contained colon mucosa samples from 12 normal people and 16 active UC patients, which was equipped with platform annotation file GPL16686. With the R language “limma” package, differential analysis was performed for screening the differentially expressed mRNAs with significance *p* < 0.05 as the screening condition.

### 2.3. Drug and Disease Common Target Acquisition

Using the jvenn tool (http://jvenn.toulouse.inra.fr/app/example.html), the above CJ-related targets were intersected with the analysis results of GSE48958 and GSE65114 datasets to obtain the drug-disease common target, the candidate target for subsequent analysis.

### 2.4. Kyoto Encyclopedia of Genes and Genomes (KEGG) Enrichment Analysis

R language “clusterProfiler” package was adopted for KEGG enrichment analysis of candidates, followed by the plotting of the full circle of KEGG enrichment analysis.

### 2.5. Visualization and Protein-Protein Interaction (PPI) Network Construction

The PPI network of candidate targets was obtained through STRING database, with the species limited to “*Homo sapiens*.” The degree values indicate the number of connections to other nodes in the regulatory network. The targets were ranked according to the degree values to show the top 15 targets. In addition, the “CJ-component-target” network diagram was completed employing the Cytoscape software (v3.8.2).

### 2.6. Immune Infiltration Analysis

Based on colon mucosal samples of 12 normal people and 16 active UC patients in the GSE65114 dataset, gene expression data were retrieved and analyzed by the CIBERSORT algorithm for the analysis of immune infiltration. Immune cell fractions were calculated in each sample, with 1000 times of computer simulations, through which only the results at the level of *p* < 0.05 were retained. CIBERSORT, a deconvolution algorithm reported by Newman et al., can characterize the cellular composition of complex tissues based on normalized gene expression profiles [18]. This method was utilized to quantify the abundance of specific cell types, which was validated by fluorescence-activated cell sorting (FACS). Gene expression data with standard annotation were uploaded to the CIBERSORT portal, and the algorithm was operated with the LM22 signature and 1,000 permutations.

### 2.7. Drug Extraction and Cell Culture

The CJ (Bozhentang Traditional Chinese Medicine, Anhui, China; http://bozhentang.1688.com/) was washed with tap water, rinsed three times with ultramorphic water, dried, and crushed. After being soaked in oil ether for 7 d for the fat removal, the drug was dried while the oil ether was filtered. Extraction was performed by solvent extraction and concentrated. It was dissolved in triple volumes of alkaline high concentration ethanol (pH = 9; 95% concentration), and the sediment was removed while supernatant was collected. The supernatant was decompressed and concentrated, and the CJ crude substance was dried at 60°C till a constant weight. The bacteria were filtered using a 0.22 *μ*m filter. Lipopolysaccharide (LPS; L2630, Sigma-Aldrich, USA) was derived from *E. coli* 0111: B4.

Mouse mononuclear macrophages RAW264.7 were procured from the American Type Culture Collection (TIB-71, USA). Cells were incubated in the Dulbecco's modified Eagle medium (11995040, Thermo Fisher Scientific, USA) appended to 10% fetal bovine serum (10099, Thermo Fisher Scientific) in 5% CO_2_ and 37°C.

### 2.8. Cell Transfection

The TLR4 CRISPR activated plasmid (sc-423419-ACT) was purchased from Santa Cruz. The RAW264.7 cells were first digested and seeded into 24-well plates to grow into a monolayer, and then, the culture medium was discarded. The transfection was performed according to the Lipofectamine 2000 instructions (11668-019, Invitrogen, Thermo Fisher Scientific, USA). Cells were transfected with TLR4 overexpression vector (oe-TLR4) or negative control (NC). After transfection, cells were cultured at 37°C and 5% CO_2_ for 6 to 8 h and then cultured in the complete medium for 24 h. Next, cells were treated with LPS (1 *μ*g/mL) or LPS combined with CJ (200 *μ*g/mL) for 24 h, after which RNA and protein were extracted for subsequent experimentations.

Based on different dosages, cells were treated with LPS (1 *μ*g/mL), low-dose CJ (50 *μ*g/mL), medium-dose CJ (100 *μ*g/mL), high-dose CJ (200 *μ*g/mL), and/or oe-TLR4.

### 2.9. Nitric Oxide (NO) Product Determination

NO generation in the culture medium was determined by the Griess method based on the nitrite accumulation. In brief, RAW264.7 cells (1 × 10^4^ cells/well) were seeded in 96-well plates and treated with LPS (1 *μ*g/mL) or LPS in combination with CJ (50, 100, or 200 *μ*g/mL) with medium alone as an NC. After 24 h of incubation, the isolated supernatant was mixed with an equal volume of the Griess reagent (G4410, Sigma-Aldrich, USA) and incubated for 20 min. The absorbance was detected at 545 nm utilizing a microplate reader (BioTek, Winooski, VT, USA). Nitrite concentrations were calculated using a standard curve, which was calculated with the help of different concentrations of NaNO_2_ solutions (0, 20 *μ*M, 40 *μ*M, 60 *μ*M, and 80 *μ*M, respectively), and the NO concentration was calculated in the light of the standard nitrite curve.

### 2.10. Enzyme-Linked Immunoassay (ELISA)

Using an ELISA kit (Beyotime, China), the TNF-*α* (PT512), IL-1*β* (PI301), and IL-6 (PI326) levels were determined in the cells. The RAW264.7 cells were seeded into 24-well plates at 2 × 10^5^ cells/well, and CJ (50, 100, and 200 *μ*g/mL) was added into cells with or without LPS (1 *μ*g/mL) for 24 h, after which supernatant was collected for determination of the TNF-*α*, IL-1*β*, and IL-6 levels.

Mouse TLR4 ELISA kit (LS-F26350, LifeSpan BioSciences, USA) was applied to detect the protein levels of TLR4 in the cells. Using the NF-*κ*B p65 (pS536+total) ELISA kit (ab176663, Abcam, UK), the protein levels of NF-*κ*B p65 and phosphorylated (p)-NF-*κ*B p65 in the cells were measured. The protein levels of JNK and p-JNK were measured in the cells using the JNK1/2 (pT183/Y185+total) ELISA kit (ab176662). Protein levels of p38, p-p38, ERK, and p-ERK were measured in the cells using the p38 MAPK alpha (pT180/Y182+total) ELISA kit (ab221013) and the ERK1 (pT202+Y204) and the ERK2 (pT185+Y187)+total ELISA kits (ab176660), respectively.

### 2.11. Reverse Transcription Quantitative Polymerase Chain Reaction (RT-qPCR)

Total RNA was extracted from cultured RAW264.7 cells utilizing the TRIzol (15596026, Invitrogen). Then, 1 *μ*g of total RNA was reverse transcribed into complementary DNA (cDNA) employing the PrimeScript™ RT reagent kit with gDNA Eraser kit (RRO37A, Takara, Japan). RT-qPCR was completed on an ABI7500 quantitative PCR instrument (Thermo, USA) applying a SYBR ®Premix Ex Taq^TM^ (Tli RNaseH Plus) kit (RR820A, Takara). With *β*-actin used as the internal reference, the 2^-*ΔΔ*Ct^ method was employed for RNA quantitation. Primers are displayed in Supplementary Table 1, which are provided by Sangon Biotech (Shanghai, China).

### 2.12. Western Blot Assay

The extracted total protein was separated by a 10% SDS-polyacrylamide gel electrophoresis and then transferred to a PVDF membrane. After blocking with 5% skim milk at room temperature, the membrane was incubated with specific antibodies of rabbit anti-CD206 (ab64693, 1 : 1000, Abcam), rabbit anti-CD11b (ab133357, 1 : 1000, Abcam), and rabbit anti-*β*-actin (#4970, 1 : 1000, CST, USA) overnight at 4°C, then incubated with secondary antibody loading buffer for 1-2 h at room temperature. The enhanced chemiluminescence solution (35050, Thermo) was dropped onto the membrane which was then exposed to the gel imaging system (ChemiDoc™ XRS; Bio-Rad, USA). *β*-Actin was used as the internal reference, and the grayscale values of the protein bands were analyzed using the “ImageJ” software.

### 2.13. UC Mouse Model Induced by Dextran Sodium Sulfate (DSS)

Thirty-six male C57BL/6 mice (20–25 g) were obtained from Beijing Vital River Laboratory Animal Technology Co., Ltd. (SCXK-2021-0011, Beijing, China). All mice were housed in the experimental animal facility for at least 1 week before the experiment and kept in plastic cages with wooden sheet bedding, with a light/dark cycle of 12/12 h at room temperature (25 ± 2°C), and freely fed with a standard diet.

Previous studies have shown that the UC mouse model can be successfully established by giving 2.5% DSS solution for 7 days [19, 20]. Based on previous literature methods, we successfully established a UC mouse model. Six randomly selected mice were given 2.5% DSS solution for drinking for 7 days, and 150 mg/kg/d 5-aminosalicylic acid (5-ASA; A3537, Sigma-Aldrich) was administered orally on 8-14 days. The rest of the mice were given 2.5% DSS solution for 7 days and were given 25, 50, and 100 mg/kg/d of CJ by gavage, respectively. Drinking water and food were provided during the experiment, and body weight, stool consistency, and rectal bleeding were recorded daily. All animal experimentations were conducted conforming to the Guide for the Care and Use of Laboratory Animals issued by National Institutes of Health and were approved by the Animal Ethics Committee of Changshu Hospital Affiliated to Nanjing University of Chinese Medicine (NJUCMCSH-AE-2021-1123).

### 2.14. Disease Activity Index (DAI)

Body weight, stool characteristics, and fecal occult blood were recorded during the experiment. The DAI was calculated in the light of the scoring system shown in Supplementary Table 2.

### 2.15. Hematoxylin-Eosin (H&E) Staining

After mice were euthanized, the colon segment (approximately 2~3 cm in length) was dissected and washed before calculation of the colon length from the cecum to the anus. Segments were then sectioned (5 *μ*m thickness) and stained with hematoxylin for 2 min and with eosin solution for 1 min. Histomorphological changes were observed under an optical microscope (XP-330, Bingyu Optical Instrument, Shanghai, China). The total damage score was determined based on the consumption of goblet cells [21].

### 2.16. Isolation of Spleen Cells

After the mice were killed, the spleen was isolated, followed by weighting of the spleen. Spleen cells were obtained through grinding [20]. Finally, the cell concentration was adjusted to 1 × 10^5^ mL using a flow buffer, which was selected for subsequent experimentations.

### 2.17. Flow Cytometry

To assess the immunomodulation of macrophage subsets in splenocytes, a standard FACS analysis was performed in the light of the protocol. CD11b was chosen to mark the M1 phenotype, and CD206 was chosen to mark the M2 phenotype. Briefly, mouse splenocytes and RAW264.7 macrophages were first incubated with an enhanced immunostaining permeabilization buffer for 8 min. Then, RAW264.7 macrophages were incubated with an antigen-presenting cell- (APC-) conjugated CD206 (MMR) and a phycoerythrin- (PE-) conjugated CD11b monoclonal antibody (M1/70, ab25482, Abcam) to evaluate macrophage subsets.

Mouse splenocytes were incubated with PE-conjugated F4/80 monoclonal antibody (ab105156, Abcam) at 4°C for 1 h and with APC-conjugated CD11b monoclonal antibody (M1/70, ab25482, Abcam) and APC-conjugated CD206 (MMR) monoclonal antibody (141707, BioLegend, USA) at 4°C for 1 h. The F4/80^+^CD11b^+^ cells were identified as M1 macrophages while F4/80^+^CD206^+^ cells were identified as M2 macrophages. Analysis was performed using a flow cytometer (Beckman, USA).

### 2.18. Immunohistochemical (IHC) Analysis

After the tissue sections were dewaxed and hydrated, the antigen was repaired with a sodium citrate buffer (0.01 M, pH = 6), and the endogenous biotin was blocked applying IHC Biotin Block Kit. Next, the tissue sections were incubated overnight with rabbit anti-CD86 (13395-1-AP, 1 : 100, Proteintech, China) and rabbit anti-IRF4 (11247-2-AP, 1 : 100, Proteintech) at 4°C. Tissue sections were then incubated with the HRP-conjugated goat anti-rabbit secondary antibody (1 : 1000, ab6721, Abcam) for 1 h, stained with a DAB immunohistochemical chromogenic kit and hematoxylin and visualized under a microscope. The average optical density value of positive color display was detected using Image-Pro Plus image analysis software. The positive protein expression was quantitatively analyzed with 5 high-fold visual field. The positive expression was confirmed as the percentage of positive cells, which was then averaged.

### 2.19. Statistical Analysis

All data were processed using the SPSS 21.0 statistical software (Armonk, NY, USA), and measurement data were expressed in the form of mean ± standard deviation. Comparisons between two groups were performed using *t*-test, and comparisons among multiple groups should be performed by one-way analysis of variance. A *p* < 0.05 indicates significant differences.

## 3. Results

### 3.1. The Potential Mechanism of CJ in Affecting UC

**Figure 1:**
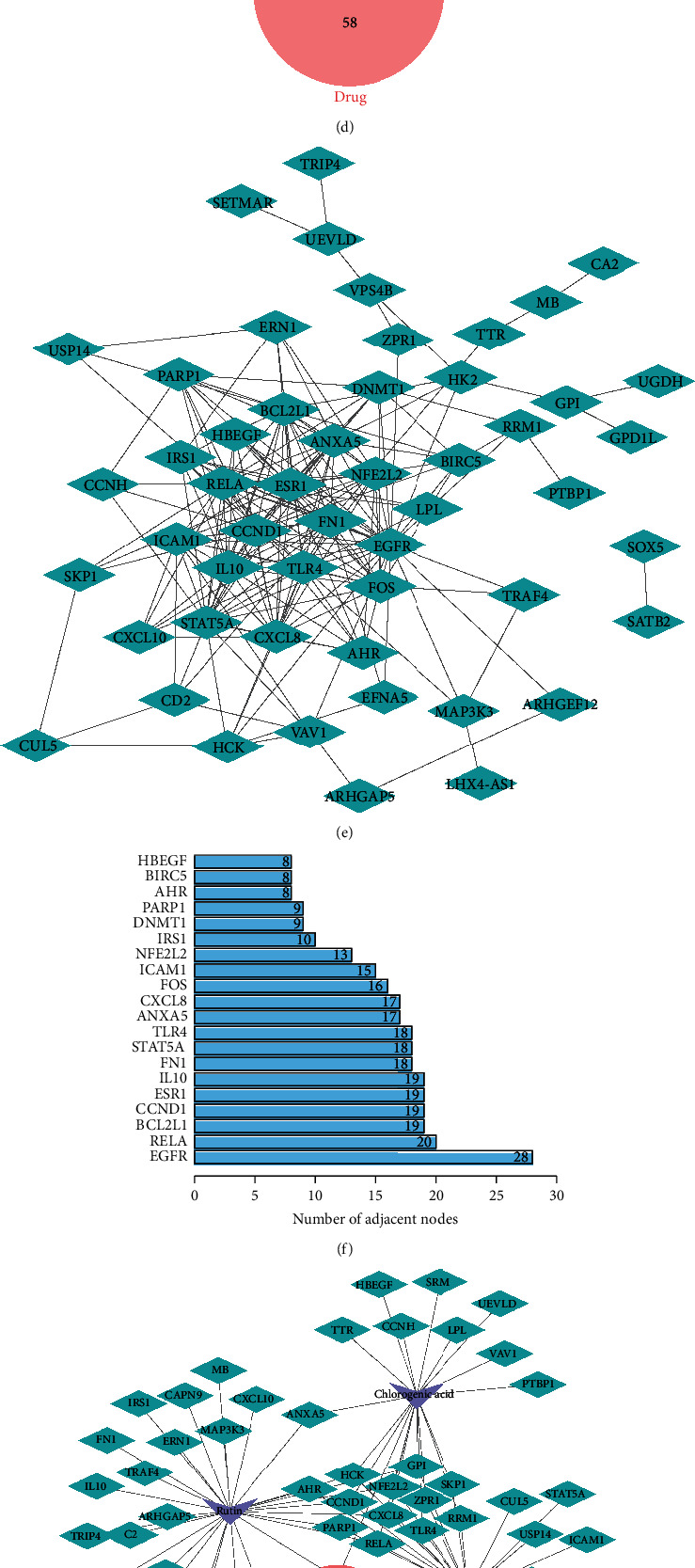
Pharmacological analysis of key targets of CJ in UC. (a) 3D pharmacophore of rutin, chlorogenic acid, and quercetin retrieved from the PubChem database. (b) Volcano map of differentially expressed targets in dataset GSE48958 (control sample = 8; UC sample = 7). Red dots represent the upregulated genes. Green dots represent downregulated genes. (c) Volcano map of the differentially expressed targets in the dataset GSE65114 (control sample = 12; UC sample = 16). (d) Venn map showing the intersection of differentially expressed targets from the datasets GSE48958 and GSE65114 and the drug targets. (e) PPI network of 60 candidate targets. (f) Top 15 targets with highest degree values displayed in the PPI network. (g) The “drug-component-target” network map. (h) KEGG enrichment analysis of the 60 candidate targets. The targets are shown in the left while the pathways are shown in the right. Counterclockwise direction starting from RELA indicates less pathway enrichment.

The main components of CJ consisted of rutin, chlorogenic acid, and quercetin. Since there is no database showing the direct targets of CJ, we predicted the targets by building the pharmacophore of the major components. Pharmacophores are models based on pharmacodynamic characteristic elements, that is, analogs with the same pharmacological effects. First, we retrieved the interactive chemical structure models of rutin, chlorogenic acid, and quercetin, respectively, through the PubChem database (Figure 1(a)) and downloaded 3D conformer and uploaded 3D conformer to the predicted targets of the PharmMapper database. Meanwhile, the corresponding targets of rutin, chlorogenic acid, and quercetin were obtained from the CTD database, and the top 50 were selected according to the interaction count ranking. Given the results of PharmMapper and CTD database retrieval, a total of 110, 111, and 99 corresponding targets of rutin, chlorogenic acid, and quercetin were obtained, respectively, with 235 drug targets in total. In addition, we obtained UC-related datasets GSE48958 and GSE65114 through the GEO database, with 11,170 and 6,739 differentially expressed targets identified using *p* < 0.05 as the threshold (Figures 1(b) and 1(c)). The drug targets were intersected with disease targets, which yielded 60 candidate targets (Figure 1(d)).

Furthermore, 60 candidate targets were imported into the STRING database for PPI analysis (Figure 1(e)), which were ranked according to the degree value. The top 15 targets were EGFR, RELA, BCL2L1, CCND1, ESR1, IL10, FN1, STAT5A, TLR4, ANXA5, CXCL8, FOS, ICAM1, NFE2L2, and IRS1 (Figure 1(f)). Based on the 60 candidate targets, the drug-component-target network map was drawn using the Cytoscape software, and the middle 12 targets (AHR, HCK, CCND1, PARP1, NFE2L2, CXCL8, RELA, GPI, ZPR1, TLR4, PRM1, and SKP1) served as common targets for at least two components (Figure 1(g)).

In addition, KEGG enrichment analysis depicted that the 60 candidate targets were mainly enriched in inflammation and infection-related pathways, as well as NF-kappa B signaling pathway and AGE-RAGE signaling pathway in diabetic complications, etc. At the same time, it could be seen that the number of pathway enrichment from high to low was RELA, FOS, and TLR4 pathway (Figure 1(h)). Combined with Figures 1(f)–1(h), the two targets, RELA and TLR4, were more critical and were the targets for multiple drug components. Rutin can block the TLR4-NF-*κ*B pathway to repress LPS-induced macrophage inflammation [12].

From what mentioned above, we hypothesized that CJ may play a role in UC by inhibiting TLR4 and its downstream NF-*κ*B/MAPK signaling pathway.

### 3.2. The Critical Role of Macrophage Polarization in UC

**Figure 2:**
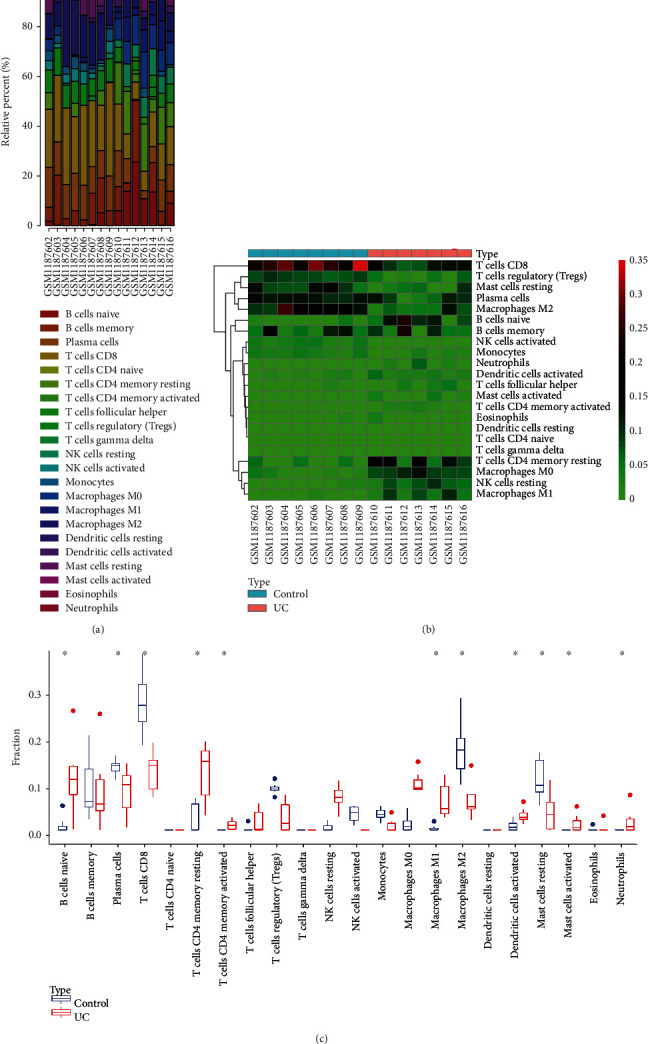
Identification of immune cell composition and differential analysis in UC samples. (a) Analysis of immune cell composition in UC samples. Abscissa indicates sample number, and ordinate indicates relative percent of immune cells. Different colors in the bar chart indicate different types of immune cells. (b) Heat map of immune cell component, wherein the abscissa indicates sample number, the ordinate indicates immune cell name, and the upper right histogram is color level. (c) Proportions of immune cell components in the control (blue) and UC (red) groups. ^∗^ indicates *p* < 0.05 versus control group.

Given the protection of inhibited macrophage polarization against DSS-induced colitis damage [22], we further conjectured whether the specific pathway by which CJ improves UC by mediating the TLR4-NF-*κ*B/MAPK pathway is associated with macrophage polarization. We analyzed the immune infiltration in the UC-related dataset GSE48958 by CIBERSORT algorithm and calculated the immune cell fractions (Figures 2(a) and 2(b)). The results showed that B cells, T cells, and macrophages were the majority of the control and UC samples, but differences were noted regarding the immune cell components. Compared with the normal samples, the proportions of M0, M1, and M2 macrophages in the UC samples were different. Further differential analysis of immune cell components in the normal and UC samples revealed the same results, with M0 macrophages and M1 macrophages having a significantly higher proportion in the UC samples, while macrophages M2 being the opposite (Figure 2(c)). The above-mentioned results depicted that macrophage polarization may play an important role in UC.

### 3.3. CJ Promotes M2 Polarization of Macrophages in DSS-Induced UC Mice

**Figure 3:**
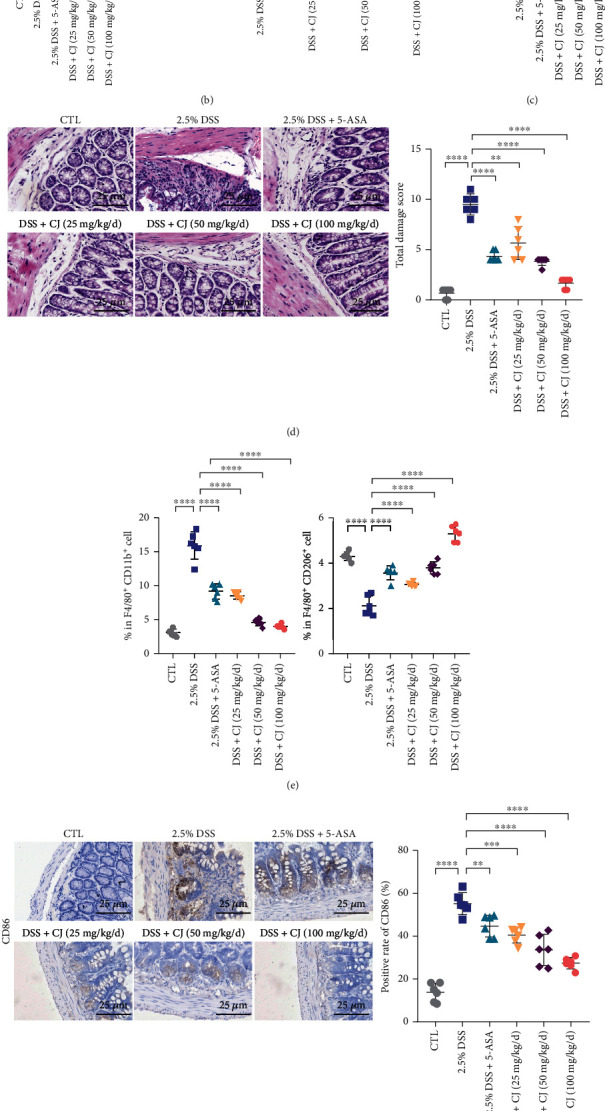
Effect of CJ on macrophage polarization in DSS-induced UC mice. (a) Loss of body weight after different administrations. (b) Colon length of mice after different administrations. (c) DAI score of mice after different administrations. (d) H&E staining for colon tissue damage after different administrations. Arrows show inflammatory infiltration, mucosal erosion, and crypt damage. (e) F4/80^+^CD11b^+^ cell and F4/80^+^CD206^+^ cell signals in the spleen shown by flow cytometer. (f) The expression of CD86 protein in the colon tissues of mice detected by immunohistochemistry. (g) The expression of IRF4 protein in the colon tissues of mice detected by immunohistochemistry. *n* = 6. ^∗^*p* < 0.05; ^∗∗^*p* < 0.01; ^∗∗∗^*p* < 0.001; ^∗∗∗∗^*p* < 0.0001.

Next, we constructed a mouse model of DSS-induced UC to explore the anti-inflammatory effects of CJ and its effects on macrophage polarization, with 5-ASA served as positive control of UC treatment. We first examined changes in body weight and colon length in mice and found reduced body weight and shortened colon length of DSS-treated mice as compared to the controls (Figures 3(a) and 3(b)). The DAI score was increased in the DSS-treated mice when compared with the controls (Figure 3(c)). Significant inflammatory cell infiltration, crypt loss, mucosal layer destruction, and edema were observed in the DSS-treated mice, corresponding to increased total damage score (Figure 3(d)). Both 5-ASA and CJ treatment inhibited the DSS-induced effects, and CJ showed a clearly dose-dependent effect. This indicated that CJ could dose dependently inhibit the occurrence of DSS-induced UC in mice.

Flow cytometric results depicted noticeably elevated F4/80^+^CD11b^+^ cells (M1 macrophages) and notably decreased F4/80^+^CD206^+^ cells (M2 macrophages) in the spleen tissues of the DSS-treated mice. Either 5-ASA or CJ inhibited DSS-induced accumulation of F4/80^+^CD11b^+^ cells, elevating the proportion of F4/80^+^CD206^+^ cells (Figure 3(e) and Supplementary Figure 1A). Detection of expression of CD86 and IRF4 in mouse colon tissues by IHC showed increased CD86 expression and decreased IRF4 expression in the DSS-treated mice, whereas reduced CD86 expression and elevated IRF4 expression were detected after 5-ASA and CJ treatment (Figures 3(f) and 3(g)).

Conclusively, CJ could inhibit DSS-induced M1 macrophage polarization in UC mice and induce M2 macrophage polarization.

### 3.4. CJ Enhances the Anti-Inflammatory Phenotype of M2 Macrophages

**Figure 4:**
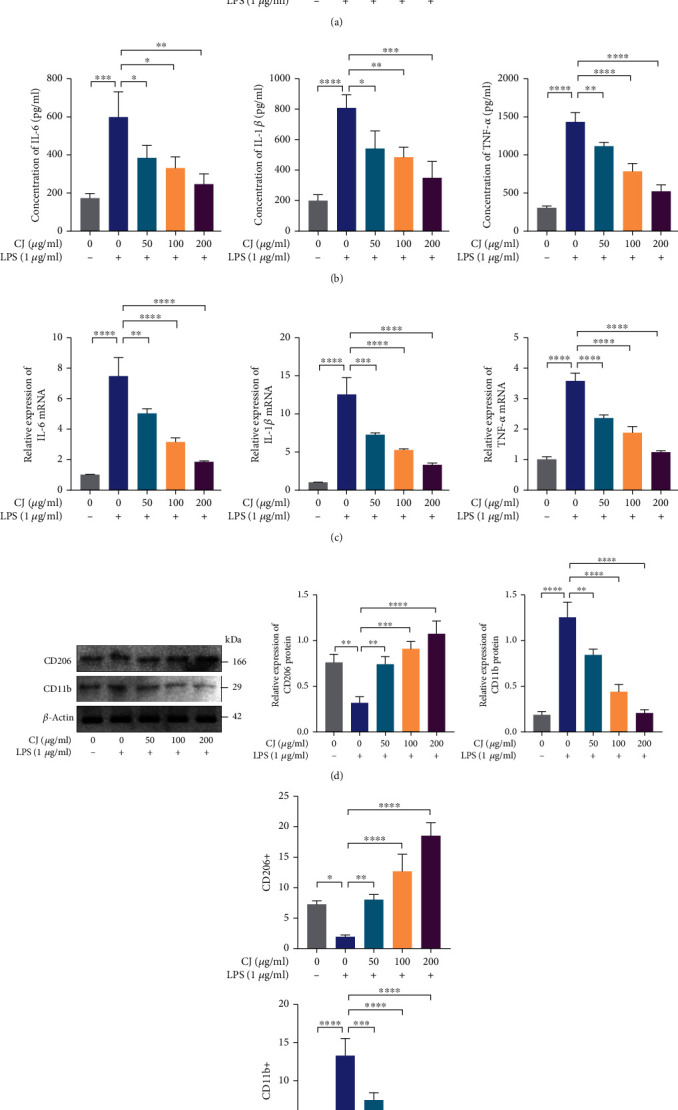
CJ inhibits the macrophage M1 polarization and enhances the macrophage M2 polarization. (a) NO concentration measured by the Griess reagent. (b) IL-6, IL-1*β*, and TNF-*α* levels in the cells detected by ELISA. (c) mRNA expression of IL-6, IL-1*β*, and TNF-*α* levels detected by RT-qPCR. (d) The protein expression of M1 macrophage markers and M2 macrophage markers in the cells measured by Western blot assay. (e) Determination of CD11b^+^ (M1 type macrophages) and CD206^+^ (M2 type macrophages) signals in RAW264.7 cells. ^∗^*p* < 0.05; ^∗∗^*p* < 0.01; ^∗∗∗^*p* < 0.001; ^∗∗∗∗^*p* < 0.0001. Cell experiments were repeated three times.

To investigate the anti-inflammatory activity of CJ exerted by promoting the M2 macrophage phenotype or suppressing the M1 phenotype, we used LPS to stimulate RAW264.7 cells and then examine the role of CJ in M1 macrophages. LPS-stimulated macrophages produced abundant NO, while the intracellular NO levels gradually decreased following treatment with CJ in concentration-dependent fashion (Figure 4(a)). LPS stimulation of macrophages increased the intracellular IL-6, IL-1*β*, and TNF-*α* levels, while CJ dose dependently reduced the levels of these inflammatory cytokines (Figure 4(b)). The mRNA expression of the inflammatory cytokines as measured by RT-qPCR was consistent with the ELISA results (Figure 4(c)). In addition, LPS increased the protein expression of the intracellular M1 macrophage marker CD11b and reduced that of M2 macrophage marker CD206; reversely, CJ treatment reduced CD11b protein expression and increased CD206 protein expression (Figure 4(d)). Furthermore, flow cytometric results depicted that LPS stimulation increased the level of CD11b-positive cells and decreased that of CD206-positive cells, while the treatment of CJ led to opposing tendency (Figure 4(e) and Supplementary Figure 1B).

In conclusion, our study suggested that CJ exerted anti-inflammatory activity by inhibiting M1 macrophage polarization and promoting M2 macrophage polarization.

### 3.5. CJ Promotes M2 Polarization to Alleviate Immune Inflammation by Inhibiting the TLR4/MAPK/NF-*κ*B Pathway

**Figure 5:**
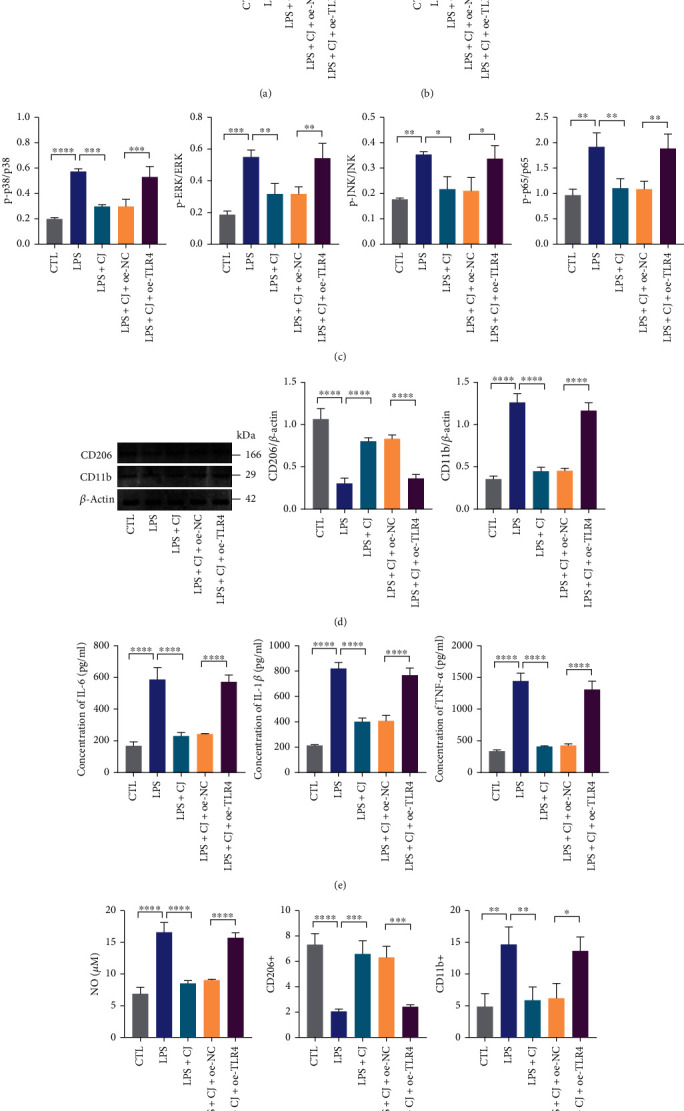
CJ affects macrophage polarization and immune activity via the TLR4/MAPK/NF-*κ*B pathway. (a) mRNA levels of TLR4 measured by RT-qPCR. LPS (1 *μ*g/mL); CJ (200 *μ*g/mL). (b) TLR4 protein expression in macrophages by ELISA. (c) Total and phosphorylated protein levels of p38, ERK, JNK, and NF-*κ*B p65 in macrophages detected by ELISA. (d) The protein expression of CD206 and CD11b in macrophages measured by Western blot assay. (e) IL-6, IL-1*β*, and TNF-*α* levels in macrophages detected by ELISA. (f) Intracellular NO concentration determined by the Griess reagent. (g) Determination of CD11b^+^ (M1 type macrophages) and CD206^+^ (M2 type macrophages) signals in RAW264.7 cells. ^∗^*p* < 0.05; ^∗∗^*p* < 0.01; ^∗∗∗^*p* < 0.001; ^∗∗∗∗^*p* < 0.0001. Cell experiments were repeated three times.

Next, we investigated whether CJ subsequently alleviated immune inflammation by regulating macrophage polarization through blockade of the TLR4/MAPK/NF-*κ*B pathway. The mRNA and protein levels of TLR4 and ratios of phosphorylated/total p38, ERK, JNK, and NF-*κ*B p65 protein were significantly increased in the macrophages following LPS treatment. CJ treatment reduced the aforementioned mRNA and protein levels and ratios of phosphorylated/total protein level in LPS-treated macrophages. Further reexpression of TLR4 enhanced the ratios of phosphorylated/total p38, ERK, JNK, and NF-*κ*B p65 protein level (Figures 5(a)–5(c)). Hence, CJ could inhibit the MAPK/NF-*κ*B pathway by inhibiting TLR4.

We also examined the expression of the CD11b and CD206 proteins. After LPS treatment, CD11b protein expression was increased in macrophages while CD206 protein expression decreased. The addition of CJ reversed LPS-induced changes in the CD11b and CD206 proteins, and further TLR4 reexpression increased protein expression of CD11b but reduced protein expression of CD206 in the presence of CJ (Figure 5(d)). Additionally, LPS treatment increased the intracellular IL-6, IL-1*β*, and TNF-*α* levels and promoted the NO generation. However, CJ treatment reduced LPS-induced production of inflammatory factors and NO, while overexpression of TLR4 attenuated the effect of CJ (Figures 5(e) and 5(f)). Furthermore, flow cytometric results depicted that LPS stimulation increased the level of CD11b-positive cells and decreased that of CD206-positive cells. However, CJ treatment reduced LPS-induced increase in CD11b-positive cells but elevated LPS-caused reduction in CD206-positive cells, while overexpression of TLR4 attenuated the effect of CJ (Figure 5(g) and Supplementary Figure 1C).

Conclusively, CJ alleviated immune inflammation by promoting M2 macrophage polarization by inhibiting the TLR4/MAPK/NF-*κ*B pathway.

### 3.6. CJ Blocks the TLR4/MAPK/NF-*κ*B Pathway in DSS-Induced UC Mice

**Figure 6:**
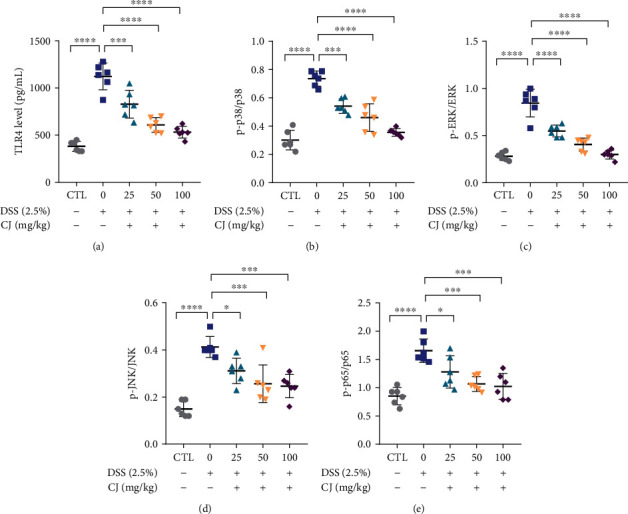
CJ regulates the TLR4/MAPK/NF-*κ*B pathway in DSS-induced UC mice. (a) TLR4 protein expression in colon tissues detected by ELISA. (b–e) The ratios of phosphorylated/total p38 (b), ERK (c), JNK (d), and NF-*κ*B p65 (e) proteins of p38 in colon tissues by ELISA. *n* = 6. ^∗^*p* < 0.05; ^∗∗^*p* < 0.01; ^∗∗∗^*p* < 0.001; ^∗∗∗∗^*p* < 0.0001.

To further validate the mechanism of action of CJ in DSS-induced UC mice, we used ELISA to examine the expression changes in the proteins relevant to the TLR4/MAPK/NF-*κ*B pathway. Results elaborated that TLR4 protein levels and the ratios of phosphorylated/total p38, ERK, JNK, and NF-*κ*B p65 protein level were enhanced in the colon tissues of the DSS-treated mice, which were dosage dependently reduced by CJ treatment (Figures 6(a)–6(e)).

Taken together, CJ could inhibit the expression of TLR4/MAPK/NF-*κ*B pathway-related proteins in the colon tissues of DSS-induced UC mice.

## 4. Discussion

DSS is often used to induce a mouse model of UC [23]. A prior study has reported that the pathogenesis of DSS-induced UC may be related to the imbalance of macrophage polarization [24]. In this study, we investigated whether and how CJ affected macrophage polarization during the development of UC using a DSS-induced UC model.

Through the component analysis of CJ, we found that the main components of CJ were rutin, chlorogenic acid, and quercetin. Rutin is a bioflavonoid existed in different fruits and vegetables, which is recognized to provide therapeutic effects in DSS-induced acute colitis [25]. Chlorogenic acid possesses potent antioxidant, antibacterial, and anti-inflammatory properties and also alleviates intestinal inflammation [26]. Quercetin has potential advantages in the treatment of inflammatory bowel diseases such as Crohn's disease (CD) and UC [27]. Our animal experiments unveiled the therapeutic effect of CJ on DSS-induced UC, evidenced by suppressed inflammatory cell infiltration and colon damage.

Furthermore, we found that CJ exerted protective functions in UC through accelerating M2 macrophage polarization. The M1 phenotype of macrophages upregulates the release of proinflammatory cytokines and chemokines and promotes the production of reactive oxygen species or nitrogen [28, 29]. Inhibition of M1 macrophage polarization contributes to reducing DSS-induced colitis damage [22]. We found that CJ increased the number of F4/80^+^CD206^+^ cells in DSS-induced UC mice, which indicated promotion of M2 macrophage polarization. As we used 5-ASA as a positive control, our data showed a similar promoting effect of 5-ASA on M2 macrophage polarization. A prior study has illustrated that administration of 5-ASA improved body weight, colon weight and length, colonic weight index, and histopathological damage in colitis mice while decreasing the levels of proinflammatory cytokines in the colonic tissues and suppressing proinflammatory macrophage activation through modulating the M1/M2 phenotype in colitis mice [30].

Our study additionally suggested that the TLR4/MAPK/NF-*κ*B signaling pathway was involved in the regulation of CJ in UC. Existing evidence identifies NFKB1 as a causal gene in the pathogenesis of inflammatory bowel diseases [31]. Inhibition of the TLR4/NF-*κ*B pathway is associated with an alleviative effect on DSS-elicited colitis [32]. Furthermore, inhibited activation of the NF-*κ*B and MAPK signaling pathways can lead to alleviated DSS-induced murine colitis [33]. The blockade of MAPK/NF-*κ*B pathway is involved in the anti-inflammatory and antioxidative functions of ginseng in DSS-induced colitis [34]. Studies have addressed the regulation of the TLR pathway by CJ components. For instance, rutin is demonstrated to decrease the expression of TLR4 [12]. Chlorogenic acid can also reverse high-fat diet-induced activation of TLR4 signaling pathway and expression of TNF-*α* and IL-6 in the liver [35]. Meanwhile, quercetin can reduce the levels of inflammatory factors in diabetic peripheral neuropathy rats via the downregulation of the TLR4/MyD88/NF-*κ*B signalling pathway [36]. Based on the above-mentioned reference, our experimental results allowed the conclusion that the TLR4/MAPK/NF-*κ*B pathway plays a contributory role in the inflammation related to UC, and CJ may alleviate UC by suppressing the TLR4/MAPK/NF-*κ*B pathway. Angiotensin 1-7 can suppress cecal ligation and puncture-induced inflammatory responses and macrophage polarization toward the M1 phenotype while promoting macrophage polarization toward the M2 phenotype via the TLR4-mediated NF-*κ*B and MAPK pathways [37], suggesting the participation of TLR4/MAPK/NF-*κ*B in macrophage polarization in the pathogenesis of UC. Our experimental results indicated that additional activation of the TLR4/MAPK/NF-*κ*B pathway could elevate the expression of M1 marker CD11b and reduce that of M2 marker CD206 in the UC mice treated with CJ, showing that CJ accelerated M2-like polarization of macrophages through blocking the TLR4/NF-*κ*B/MAPK pathway.

## 5. Conclusion

**Figure 7:**
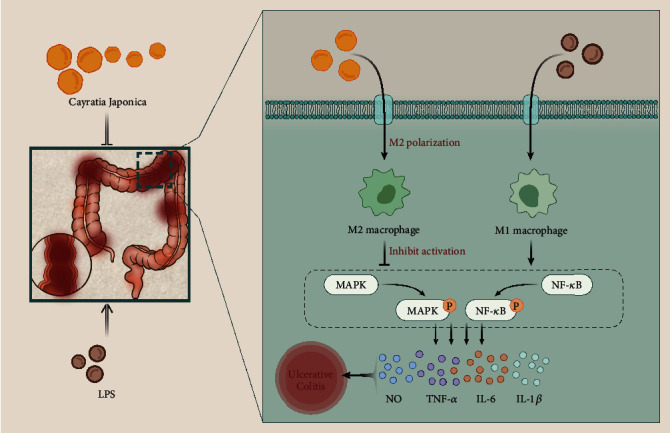
Schematic representation of the molecular mechanisms by which CJ regulates macrophage polarization affecting UC development via the TLR4/MAPK/NF-*κ*B pathway.

Collectively, our work underpinned that CJ suppressed the inflammatory responses in RAW 264.7 macrophages and in established UC animal models, probably through blockage of the TLR4/NF-*κ*B/MAPK pathway by inhibiting M1 macrophage polarization and promoting M2 macrophage polarization (Figure 7). This study suggests a promising therapeutic strategy based on CJ for UC treatment.

## Data Availability

The datasets used and/or analyzed during the current study are available from the corresponding authors on reasonable request.

## Supplementary Materials

Supplementary Materials Supplementary Figure 1: representative images for Figures 3(e), 4(e), and 5(g). Supplementary Table 1: primer sequence for RT-qPCR. Supplementary Table 2: DAI scoring system.
