# Supplementary material for: Cayratia japonica Prevents Ulcerative Colitis by Promoting M2 Macrophage Polarization through Blocking the TLR4/MAPK/NF-κB Pathway
Source: Mediators Inflamm. 2022 Dec 30;2022:1108569. doi: 10.1155/2022/1108569 (PMC9822765; doi:10.1155/2022/1108569)
Supplement: Supplementary Materials — Supplementary Figure 1: representative images for Figures 3(e), 4(e), and 5(g). Supplementary Table 1: primer sequence for RT-qPCR. Supplementary Table 2: DAI scoring system. [file 1108569.f1.zip › Supplemental Files (1).docx]

Supplementary Table 1 Primer sequence for RT-qPCR

| Target | Sequence |
| --- | --- |
| TNF-α | F: 5’-TGTCCCTTTCACTCACTGGC-3’ |
|  | R: 5’-CATCTTTTGGGGGAGTGCCT-3’ |
| IL-1β | F: 5’-CCCAACTGGTACATCAGCAC-3’ |
|  | R: 5’-TCTGCTCATTCACGAAAAGG-3’ |
| IL-6 | F: 5’-CCACTTCACAAGTCGGAGGCTTA-3’ |
|  | R: 5’-GCAAGTGCATCATCGTTGTTCATAC-3’ |
| TLR4 | F: 5’-ACACCAGGAAGCTTGAATCCC-3’ |
|  | R: 5’-CATCAGGGACTTTGCTGAGTT-3’ |
| β-actin | F: 5’-AGAGGGAAATCGTGCGTGAC-3’ |
|  | R: 5’-CAATAGTGATGACCTGGCCGT-3’ |

Note: RT-qPCR, reverse transcription-quantitative polymerase chain reaction; F, forward; R, reverse.

Supplementary Table 2 DAI scoring system

| Score | Weight loss (%) | Stool character | Fecal occult blood |
| --- | --- | --- | --- |
| 0 | 0 | Normal formed | Negative |
| 1 | 1-5% |  |  |
| 2 | 5-10% | Loose stool | Positive |
| 3 | 10-20% |  |  |
| 4 | ＞20% | Diarrhea | Gross bleeding |

Note: DAI, disease activity index.

**Supplementary Figure 1** A, Representative images of figure 3E. B, Representative images of figure 4E. C, Representative images of figure 5G.
